# Hiding in plain sight: communication theory in implementation science

**DOI:** 10.1186/s13012-015-0244-y

**Published:** 2015-04-23

**Authors:** Milisa Manojlovich, Janet E Squires, Barbara Davies, Ian D Graham

**Affiliations:** University of Michigan School of Nursing, 400 N. Ingalls, room 4306, Ann Arbor, MI 48109 USA; School of Nursing, University of Ottawa, Ottawa, Canada; Clinical Epidemiology Program, Ottawa Hospital Research Institute, Ottawa, Canada; Epidemiology and Community Medicine, University of Ottawa, Ottawa, Canada; Centre for Practice-Changing Research, Ottawa Hospital Research Institute, Ottawa, Canada

**Keywords:** Communication, Physician-nurse relations, Theoretical models, Knowledge translation

## Abstract

**Background:**

Poor communication among healthcare professionals is a pressing problem, contributing to widespread barriers to patient safety. The word “communication” means to share or make common. In the literature, two communication paradigms dominate: (1) communication as a transactional process responsible for information exchange, and (2) communication as a transformational process responsible for causing change. Implementation science has focused on information exchange attributes while largely ignoring transformational attributes of communication. In this paper, we debate the merits of encompassing both paradigms.

**Discussion:**

We conducted a two-staged literature review searching for the concept of communication in implementation science to understand how communication is conceptualized. Twenty-seven theories, models, or frameworks were identified; only Rogers’ Diffusion of Innovations theory provides a definition of communication and includes both communication paradigms. Most models (notable exceptions include Diffusion of Innovations, The Ottawa Model of Research Use, and Normalization Process Theory) describe communication as a transactional process. But thinking of communication solely as information transfer or exchange misrepresents reality. We recommend that implementation science theories (1) propose and test the concept of shared understanding when describing communication, (2) acknowledge that communication is multi-layered, identify at least a few layers, and posit how identified layers might affect the development of shared understanding, (3) acknowledge that communication occurs in a social context, providing a frame of reference for both individuals and groups, (4) acknowledge the unpredictability of communication (and healthcare processes in general), and (5) engage with and draw on work done by communication theorists.

**Summary:**

Implementation science literature has conceptualized communication as a transactional process (when communication has been mentioned at all), thereby ignoring a key contributor to implementation intervention success. When conceptualized as a transformational process, the focus of communication moves to shared understanding and is grounded in human interactions and the way we go about constructing knowledge. Instead of hiding in plain sight, we suggest explicitly acknowledging the role that communication plays in our implementation efforts. By using both paradigms, we can investigate when communication facilitates implementation, when it does not, and how to improve it so that our implementation and clinical interventions are embraced by clinicians and patients alike.

## Background

Poor communication among healthcare professionals is a pressing problem, contributing to widespread barriers to patient safety [1]. For example, poor communication between physicians and nurses is one of the most common causes of adverse events for hospitalized patients [2-4] and a major root cause of all sentinel events [5]. Nurses—as the 24-h surveillance system for hospitalized patients—are often the first to detect early signs of patient deterioration [6,7] and, in raising the alarm, must communicate in a way that physicians will understand, because information must be understood before it can be acted upon [8]. The word “communication” has its roots in Latin, “*communicare*,” meaning to share or make common [9]. Two communication paradigms, described in Table 1, dominate the literature: (1) communication as a transactional process responsible for information exchange, and (2) communication as a transformational process responsible for causing change [10].

**Table 1:** **Communication paradigms** [1]

**Communication as an information exchange**	**Communication as an interpersonal process**
Definition: the process by which information is exchanged between individuals or computers through the use of a commonly accepted set of symbols.	Definition: a process of developing shared understanding by establishing, testing, and maintaining relationships.
Transactional—focus is on transfer of information	Transformational—focus is on changes as a result of communication
Improvement in communication occurs through standardization of information	Improvement in communication occurs through interpersonal relationships
Environment does not play a central role in communication	Organizational complexity (i.e., environment) is one of three dimensions (along with social context and cognitive load) recognized as influencing communication

In this paper, we debate the merits of encompassing both paradigms of communication in the implementation science literature. We argue that implementation science should adopt a definition of communication that hews more closely to its Latin roots: communication as a process of developing shared understanding which emerges by establishing, testing, and maintaining relationships between communicators [11]. Implementation science refers to “the scientific study of methods to promote the systematic uptake of clinical research findings and other evidence-based practices into routine practice” [12]. Given that the purpose of implementation is to facilitate the uptake of evidence, placing an emphasis on actions instead of words by incorporating the above definition of communication may help move the field of implementation science forward. The following example illustrates why a debate on this topic is needed. We use an example from clinical practice to provide the context for our debate because despite robust evidence from more than one randomized control trial evidence-based practices known to improve outcomes for mechanically ventilated patients are not implemented routinely. This suggests that more robust theory is needed to address the implementation challenges found in clinical practice, so that we can select implementation interventions with potentially stronger effects [13].

Mechanically ventilated patients who receive continuous sedative infusions have been found, in two randomized controlled trials, to benefit from a multi-disciplinary clinical intervention with implications for implementation [14,15]. The daily interruption of sedation is an evidence-based intervention, [16] requiring the application of new knowledge (i.e., identifying which patients are candidates for the intervention, the sequence of steps needed to carry out the intervention, assessing patient effect). The daily interruption of sedatives is a multi-disciplinary intervention because the sedative order comes from medicine, the sedative is dispensed by pharmacy, stopping the sedative infusion is carried out by nursing, and assessment of the effects of the intervention is jointly made by nursing and respiratory therapy, who then report to medicine for additional orders, if needed. Despite significant benefits to patients (i.e., decreasing the number of ventilator days, reducing mortality, and limiting risks of ventilator-associated events [14,17]) and a sound evidence base, daily interruption of sedation is implemented by fewer than 50% of ICU healthcare professionals [18]. Miller and colleagues [19] set out to gain greater understanding of the reduced implementation of this intervention. Through focus groups of ICU physicians and separate groups of nurses and respiratory therapists, the investigators found that shared understanding of the need to carry out daily interruption of sedation was missing, representing failure of the implementation intervention which contributed to inconsistency in the type of patients who were selected (thus fewer may have been selected than were eligible) as well as different approaches to how the intervention was carried out. Miller and colleagues concluded that “little attention has been paid in the literature to communication of the medical goals and fundamental mechanisms of the intervention itself, thus highlighting a potentially important area for implementation science” (p. 281.e5) [19].

This example demonstrates that the manner in which a multi-disciplinary intervention is communicated (in the sense of developing shared understanding) is an activity fraught with many challenges, [1] yet one for which there is little guidance. Each healthcare discipline brings its own distinct knowledge base to bear on a clinical situation [20]. Complex or unique problems, such as those often encountered in hospital settings, require knowledge building as part of the solution [21]. However, knowledge building in healthcare comes from disciplines trained in separate spheres and paradigms, [22] requiring a bridge we have not yet succeeded in building. We believe that knowledge is socially constructed, meaning that people create knowledge through their interactions, [23] mostly accomplished via communication.

### The social construction of knowledge

We take a sociological view of the concept of communication, using the lens of social constructionism, [23] which emphasizes “purposeful creation of knowledge” [24]. This view is in contrast to a similar concept, social constructivism, which refers to knowledge creation by the individual [24]. Groups of individuals through their interactions create a social reality which is an ongoing, dynamic process with individuals acting on their interpretation of the perceived social reality [24]. Two key concepts to a social constructionism perspective are that the environment or social context is incorporated into knowledge building and that the group’s attention is on knowledge which is jointly created [24]. These key concepts are important since human activity in any group tends to fall into patterns and routines which form the reality of everyday life [23]. Patterns and routines contribute to the creation of social phenomena which are then institutionalized so that a negotiated order emerges, one to which all group members subscribe either implicitly or explicitly, forming the culture of that group [24]. Implementation research methods implicitly use a social constructionist lens when they recognize the importance of group dynamics and culture; participatory action research is one example. We are advocating for the same social constructionist lens to be applied to the concept of communication, to explicitly call out the role of the group situated in a specific context in developing the shared understanding necessary for implementation intervention success.

Next we discuss the results of a literature search done to identify how communication is defined and conceptualized in implementation science theories. Then we articulate the need for a multi-dimensional concept of communication in implementation science, one that draws from both communication paradigms and places communication in the context of the social construction of knowledge. We conclude with several recommendations for moving implementation science forward by purposefully expanding the concept of communication to include the notion of communication as developing shared understanding.

## Discussion

### Literature review

We conducted a two-staged literature review searching for the concept of communication and its use in implementation science. We began with a review of known implementation models and then moved to a broader scoping review of the literature. We searched a variety of sources for the review of implementation models: the Consolidated Framework for Implementation Research (CFIR), [25] a thematic analysis of 28 models conducted by Ward and colleagues, [26] a review of 61 implementation and dissemination theories conducted by Tabak and colleagues, [27] and an in-depth review of 8 evidence-based practice models and frameworks [28].

The CFIR is a comprehensive typology of common constructs found in the published literature of 19 implementation theories [25]. The theories chosen for inclusion in the CFIR were derived from the systematic literature review of implementation theories done by Greenhalgh in 2004 [29]. The CFIR acknowledges the importance of relationships (as part of networks) to implementation but views communication as separate from networks. The CFIR lacks detail on specific attributes of communication (other than “high quality”), but given that the CFIR is a meta-theory, it is understandable that the granular level of detail needed to illuminate communication as a key concept is missing. We retrieved and closely examined the 12 articles identified in the CFIR that included communication as a construct, and these are the first 12 articles displayed in Table 2 which identifies implementation models in which the need for effective interdisciplinary communication is either implied or appears as an explicit concept.

**Table 2:** **The concept of communication in implementation models**

**Implementation model**	**Communication: explicit or implied?**	**How communication is conceptualized and/or defined**	**Theoretical underpinnings of the model**
Dimensions of strategic change [68]	Explicit	Communication mechanisms are mentioned, but there is no definition.	Competition and strategic change
A multi-level conceptual framework of organizational innovation adoption [69]	Explicit	Communication is conceptualized in several ways: as a marketing activity done by suppliers to influence potential customers’ perceptions; as a medium (i.e., communication technology or system); as an interpersonal process. There is no definition.	Diffusion of innovations
A conceptual model for implementation effectiveness [37]	Explicit	Implementation climate instrument includes six items to measure communication, conceptualized as information exchange. There is no definition.	Organizational behavior
Promoting Action on Research Implementation in Health Services (PARIHS) [35]	Implied	Communication is inferred in the element of context.	Diffusion of innovations; organizational theories and humanism
A conceptual framework for transferring research to practice [70]	Explicit	Communication is mentioned as an element of the climate in which change is to occur, but not defined.	Organizational behavior; diffusion of innovations
A conceptual model for considering the determinants of diffusion, dissemination, and implementation of innovations in health service delivery and organization [29]	Explicit	Several views of communication are provided. Mention is made of communication channels; interpersonal and inter-organizational communication; communication as a component of the diffusion process. There is no definition.	Diffusion of innovations
Ottawa model of research use [50]	Implied	Communication is implied in several stages of the model and conceptualized as interpersonal process (e.g., adapting knowledge requires dialogue; lack of mutual understanding between disciplines is a barrier to knowledge use).	Diffusion of innovations; planned action theory
Availability, responsiveness, and continuity: An organizational and community intervention model [39]	Explicit	Change agents are charged with facilitating communication, but no definition of communication is provided.	General systems theory; diffusion of innovations; socio-technical theory; organizational theories
An organizational transformation model [71]	Explicit	Communication is identified as a factor necessary for successful change, but not defined.	Microsystems; diffusion of innovations
Will it work here? A decision-maker’s guide adopting innovations [72]	Explicit	Communication is conceptualized in several ways: as information exchange; as an outcome (e.g., improved communication); as a skill; also, “bridge communication gaps.” There is no definition.	Diffusion of innovations
A practical, robust implementation and sustainability model (PRISM) [51]	Explicit	Communication is conceptualized in two ways: as a bridge between researchers and adopters, and as a managerial activity to help convey sense of support. There is no definition.	Diffusion of innovations; social ecology; chronic care model
A framework of dissemination in health services intervention research [73]	Implied	Communication is inferred by phrases such as “networks and linkages” and “flows of information.”	Social cognitive and learning theories; organization and social change theories; agency theory; diffusion of innovations
A conceptual framework for transferring knowledge into action [26]	Explicit	Communication is mentioned as a common component of the knowledge transfer process, and conceptualized as information transfer, but not defined.	Framework drawn from multiple theories
Normalization process theory (NPT) [33]	Implied	Communication is implicit in this theory which has interaction and group processes as foundational elements.	Sociological theories focusing on social processes
An organizational theory of implementation effectiveness [36]	Explicit	“Persuasive” communication is mentioned but not defined.	Organizational behavior
A model for large-scale knowledge translation [34]	Implied	Engaging, educating, executing, and evaluating interventions require communication.	Not evident
Sticky knowledge [38]	Explicit	Communication theory is mentioned, and a link between knowledge transfer and communication is described (i.e., ease of communication). Communication gaps between the source and recipient of knowledge are mentioned. There is no definition.	Communication theory; strategic management
A conceptual model of evidence-based practice implementation [40]	Explicit	Communication is described in two ways: as a product, and as pathways. There is no definition.	Diffusion of innovations
Stetler model [28]	Implied	Communication is implied in the group facilitation required for research utilization.	Planned action theories
Iowa model of evidence-based practice [28]	Implied	An assumption of the model is that working as a group is an important part of applying evidence in practice, which suggested the use of communication.	Quality and performance improvement, organization and systems literatures
Dissemination and use of research evidence for policy and practice [28]	Implied	The model describes a process by which “decision makers engage in evidence-based decision making”, implying the use of communication.	Diffusion of innovations
Advancing research and clinical practice through close collaboration [28]	Implied	Collaboration with interdisciplinary professionals to foster evidence-based practices implies the use of communication.	Control theory and cognitive behavior theory
The Joanna Briggs Institute model of evidence-based healthcare [28]	Implied	Evidence or knowledge transfer requires communication.	Not evident
The Knowledge-to-Action framework [28]	Implied	A key mechanism for turning knowledge into action is social interaction, which implies the use of communication.	Planned action theories
The Quality Implementation Framework (QIF) [47]	Explicit	“Effective” communication is mentioned but not defined.	Diffusion of innovations
The Tehran University of Medical Sciences Knowledge translation Cycle [48]	Explicit	Communication between producers and users of knowledge is mentioned; communication is identified as a skill and as a network. There is no definition.	Not evident
Diffusion of innovations [49]	Explicit	Communication is defined as a process in which participants create and share information to reach mutual understanding.	Sociological theory

Ward and colleagues conducted a thematic analysis of the knowledge translation literature and identified 28 different models that to varying degrees explain the knowledge translation (or implementation) process [26]. Communication was coupled with problem identification as a common component of knowledge translation, but in the conceptual framework developed by the authors, the word “communication” is missing.

Tabak and colleagues reviewed 61 theoretical models that focused on dissemination and/or implementation activities [27]. Since our focus is on implementation, we restricted our examination to implementation models only, although we found (as did Tabak) considerable overlap between models. Of the 12 implementation models identified by Tabak, three make no mention of communication, [30-32] communication is implied in three models, [33-35] and explicitly described (but not defined) in the remaining six models [25,36-40]. Models mentioning communication, but not already in the CFIR, are added to Table 2. The notion that communication might refer to the development of shared understanding was not included in any of the six models in which communication was described.

Rycroft-Malone and Bucknall [28] provided an in-depth analysis of eight theoretical models for implementing evidence-based practice. Models were chosen for analysis based on six explicit criteria: international recognition, subject to evaluation and/or testing by someone other than authors, transferable across settings, models sampled from different disciplines, new and established models included, and model developer willing to author a chapter in the book using a standardized template to facilitate model synthesis [28]. Of the eight models, two are already in Table 2 (the Ottawa Model of Research Use (OMRU) and Promoting Action on Research Implementation in Health Services (PARIHS) models). The remaining six models do not explicitly include the term communication, although it is implied, and are added to Table 2 [41-46].

In the second stage, we conducted a scoping review to make sure that we did not miss relevant theories and to provide an update since 2009 when the CFIR was published. We searched PubMed and CINAHL databases using the terms: “implementation theory”, “implementation model,” “implementation framework,” “knowledge translation theory”, “knowledge translation model”, and “knowledge translation framework.” Again, inclusion criteria were papers reporting on implementation theories or models; dissemination and combined implementation/dissemination models were excluded. This search yielded an additional two theories not previously identified: the Quality Implementation Framework [47] and the Tehran University of Medical Sciences knowledge translation cycle [48]. Our search strategy did not capture the groundbreaking work of Everett Rogers as described in the *Diffusion of Innovations* [49]. However the influence that Rogers had on the field of implementation science is obvious by the number of models in the table derived from Rogers’ work, and so Rogers’ framework is included in Table 2.

In total, 27 theories, models, or frameworks were identified across all searches. The field of implementation science, or knowledge translation as it is also known, [46] has tended to focus on the information exchange attributes of communication while largely ignoring how shared understanding develops. The notion that communication includes developing shared understanding is generally absent; the Ottawa Model of Research Use [50] was the only model to conceptualize communication as an interpersonal process and acknowledge that a lack of shared understanding is a barrier to knowledge use. We found inconsistent use of the term communication across or even within theories. For example, in the PRISM model, communication has two meanings: as a way to connect researchers and adopters and as a managerial activity that conveys support [51]. In the conceptual model of evidence-based practice implementation, communication refers to an informal pathway and also to a product (e.g., send out “communications”) [40].

In summary, only Rogers’ Diffusion of Innovations theory [49] provides a definition of communication, consistent with the term’s Latin roots. No other theory, model, or framework defined communication. Interestingly, although the Diffusion of Innovations theory undergirds most implementation theories conceptually, none have adopted Rogers’ definition of communication. Most models (notable exceptions include Diffusion of Innovations, The Ottawa Model of Research Use, and Normalization Process Theory) lean towards understanding communication as a process by which information is exchanged and disseminated as part of an implementation intervention. While this view of communication is necessary for implementation science, it is not sufficient because it assumes that the information is understood by all parties taking part in the implementation process. We also found that there was no consistency between theoretical underpinnings common to many models (diffusion of innovations or organizational behavior, for example) and how communication was conceptualized.

In our search of the literature, there was only one instance where a definition of communication as the development of shared understanding was used [49]. Yet this definition is important to implementation science because knowledge use will be hindered unless a way is found to bridge diverse perspectives or find consensus among them. Lack of conceptual coherence or definition leads us to conclude that communication is “hiding” in implementation theories. A multi-dimensional view of communication is needed, one that acknowledges the social nature of human activities which helps us understand that communication is more than information transfer and that humans have to develop shared understanding through communication to function in groups. We argue that both paradigms are needed. The implementation science literature, with its focus on the uptake of evidence into practice, largely does not address how shared understanding develops so that new knowledge can be applied effectively across disciplines.

### A range of approaches in communication research

There are a wide variety of approaches currently in use in human communication research, depending on whether the interest is on a specific type of communication or on a process associated with communication. Types of communication such as conversation, interviewing, and public speaking have frequently been the focus of communication research. For example, conversation analysis (or CA) considers utterances as social activities and closely examines the sequencing of utterances to provide information on features of the social context [52]. In CA, utterances are “social objects that accomplish actions” [52] and as such have great utility for implementation science. CA might be used to determine the success of an implementation intervention because not only could CA identify ongoing barriers to a specific intervention but perhaps more importantly reveal why those barriers persist.

Examples of research on communication as a process include cognition and information processing and social construction through interpersonal processes. We believe that the notion of communication as an interpersonal process has not received sufficient attention and this may be one contributing factor to the lack of success of some implementation interventions. Viewing communication as a process of developing shared understanding puts the focus of communication more on the outcome—the action—arising from a communication exchange rather than on the content of the message itself [10]. Without such an emphasis, there is little understanding of how communication can contribute to knowledge use or even knowledge development which is needed for successful implementation. Many communication processes are so embedded into the structure of work that they are invisible, and the relationship between communication and action has been lost [53]. For example, on a hospital inpatient unit, an “x” on a white board beside a patient’s name may indicate that the patient is scheduled for discharge and as such is a form of communication aimed at clerk, nurse, and others who must have a shared understanding of what the “x” means before the incipient discharge becomes an action [53]. All communication processes share this link between communication and action because through them we make sense of and change our world.

Examining the role of conversation in communication can take more than one form. CA, described above, focuses on utterances, but other researchers have taken a more global approach. As Parker and Coiera maintain, “It is through the multitude of conversations that pepper the clinical day that clinicians examine, present, and interpret clinical data and ultimately decide on clinical actions” [54]. Similarly, it is through these conversations—or communication—that clinical as well as implementation interventions occur [55]. Conversations between healthcare providers may contribute to variation in clinical outcomes, and intervention success may depend on whether or not communication is deliberately built into the intervention design, “regardless of the nature or scope of the intervention” [55]. Conversations are jointly constructed and involve three concepts: collaboration, sensemaking, and improvisation [55]. In order to understand and be understood by others, participants in a conversation make an implicit agreement to collaborate or else the series of conversational turns that make up a conversation will stop: a verbal comment or non-verbal signal will mark the end of a conversation without collaboration between participants [55]. Sensemaking emerges from the joint construction of a conversation because no part of conversational turns (i.e., content, sequence, allocation to participants) is pre-specified or can be predicted [55]. Through conversations, old beliefs can be reinforced and strengthened or innovative new ideas can emerge for the first time [55]. Sensemaking may be an especially important development; intervention success may depend on sensemaking because through the conversation the meaning is “narrowed or broadened, and options are selected, clarified, reduced, added or created” [55]. Thus joint agreement on the meaning is what contributes to intervention success [55]. Finally, understanding among conversation participants is enhanced through the final concept that characterizes conversations: improvisation. Although some aspects of conversation can be scripted by using a communication tool such as Situation, Background, Assessment, Recommendation (SBAR), every conversation is unique and unpredictable because participants improvise, acting from moment to moment rather than following conversation rules rigidly [55]. Other researchers have developed theoretical frameworks to explain how relationship building through communication contributes to healthcare quality [10,56]. For example, Lanham and colleagues developed a conceptual model of the relationship between primary practice characteristics and outcomes at the practice level. They conducted a secondary data analysis of four studies to understand the characteristics of relationships in over 200 primary practice settings and described how relationships contributed to a range of practice improvement success [56]. Findings suggest that those primary care practices that exhibited characteristics such as “respectful interaction” and “social/task relatedness” (among others) as well as effective communication were more successful in their practice improvement efforts [56].

Pirnejad and colleagues described two general conceptual frameworks to explain how communication can be improved in healthcare [10]. In the first conceptual framework, common in medical informatics, the communication space is part of the healthcare information space, with the focus on information exchange [10]. In the second framework, common in cognitive and social sciences, communication is viewed as larger than the healthcare information space because every communication exchange has a social dimension which plays a crucial role in understanding the central message [10]. According to the second framework, healthcare is provided in a social environment [57] which provides the context for interactions between people. Jacobi describes four characteristics of communication that may help us understand how communication as an interpersonal process may contribute to greater implementation intervention success [9]. Like Pirnejad [10] and others [3,54,55,58] who turn a sociological lens on the study of communication, Jacobi asserts that communication is relational, unlike the sender-receiver model of communication in which the message always comes prior to the receiver [9]. In the sender-receiver model, the content of a message must be taken at face value by the receiver without an opportunity for the receiver to put the message in context and reflect on it [9]. Without the back-and-forth, iterative dialogue needed to assure that a message is not only received but understood by all parties, nuances or even key points may be missed that can hamper implementation intervention success.

Second, Jacobi maintains that communication is multi-layered coming forth from various layers of meaning which can complement or contradict each other [9]. Because of the multiple layers of meaning, different actors can join in a communication interaction, and as a result, each actor can choose a different meaning as a point of entry. The multiple layers of meaning make communication ambiguous, but the ambiguity is viewed as a “surplus of truth,” [9] with implications for multi-disciplinary teams, since each discipline approaches a communication exchange from its own “truth.” The many common areas of knowledge between healthcare disciplines can sometimes mask the fact that each discipline also has its own “truth” or distinct knowledge base. For example, a patient with pneumonia newly admitted to the hospital needs medical care, nursing care, and respiratory therapy care at a minimum. The patient has an abnormally high heart rate and tells the nurse that he is anxious about being in the hospital and missing work, so the nurse views the high heart rate as a sign of anxiety and considers nursing interventions to reduce it. In the meantime, the respiratory therapist looks at the high heart rate on the bedside monitor and attributes it to the inhaler she just administered. The physician looks at the high heart rate and wonders if the patient has a fever. The same physiological parameter (heart rate) has dissimilar meanings depending on the discipline of the healthcare provider who is viewing it, and without communication, interpretations of the meaning of the high heart rate may differ.

Third, according to Jacobi, communication is “a continuous process of (re-) contextualization rather than a loose sequence of *ad hoc* interactions” [9]. Thus communication is contextual, both in a social sense as well as in an individual sense [9]. Socially, any communication is situated within some frame of reference known to those involved in the exchange, whether that frame of reference refers to an event, a topic, an activity, or something else. Individually, each communicator brings a set of values which are implicit or explicit, expressed or suppressed. These values contribute to and anchor the frame of reference to provide a unique contextual foundation for each communication episode. Understanding the contextual nature of a communication exchange is important to implementation intervention success and knowledge translation efforts because without appropriate context an intervention may drift away from its original intent [59].

Finally, Jacobi asserts that communication is more than language embodied in face-to-face contacts, and indeed up to 80% of communication may be non-verbal [60]. Jacobi acknowledges that other media are needed to disseminate the knowledge gained through communication beyond those directly involved in the communication exchange [9]. Face-to-face and telephone exchanges represent the use of rich media because they capture multiple channels at once (e.g., visual, auditory) whereas pagers and other communication technologies that deliver text messages represent the use of less rich media because they capture only one channel [61]. Media richness is defined as a characteristic of a communication medium that facilitates the ability of information being sent through that medium to change understanding [61]. Classification is based on a medium’s capacity for immediate feedback, the number of cues and channels used, personalization, and language variety [61]. Including consideration of the medium used for communication purposes may be an important component to intervention success, especially if the intervention is complex and requires understanding from multiple disciplines, as in our example above.

### Moving forward

How can our view of communication be incorporated into current and emerging implementation theories? There are many different methods of theory development as well as theory derivation which stand out in the implementation literature. For example, Graham and Logan suggested the application of theoretical pluralism for how additional theories might be added to the OMRU, a conceptual framework grounded in knowledge theory [50]. The OMRU contains generic concepts such as innovation, adopters, and practice environment all of which could be enhanced by additional theories, depending on which of these concepts the theory was designed to address. Using theoretical pluralism for example, a micro- or practice-range communication theory could be embedded under the “adopters” or the “practice environment” concepts.

“Knowledge translation” is the term commonly used in Canada to refer to implementation [46]. The knowledge translation movement is moving towards integrated knowledge translation, which uses a collaborative approach to focus on engagement with the users of the research and the context in which they work. Researchers and stakeholders collaborate on all phases of the research to improve the likelihood that results will be applicable to the population being studied. An integrated knowledge translation paradigm, with its emphasis on the co-creation of knowledge, also has its roots in sociology. Previous literature has focused on identifying key concepts and evidence that can be used to guide implementation activities such as identifying target audiences and assessing barriers and facilitators to proposed implementation strategies [62]. The shift to integrated knowledge translation represents an evolution in implementation science and an opportunity to incorporate a broader view of communication.

We have five core recommendations for moving forward the notion of communication as developing shared understanding in the implementation science literature. Our recommendations are not in any particular order because we believe that, depending on the research question and implementation theory being used, one recommendation may need to be prioritized over another.

First, implementation science theories should propose and test the concept of shared understanding when describing communication and acknowledge the relational nature of communication. In a social constructionist paradigm, knowledge emerges from joint interactions or communication and thus depends on relationships, which are necessary to facilitate understanding of varying perspectives [63]. Researchers would benefit from implementation theories that propose some plausible paths by which shared understanding might be obtained. Through testing of these theoretical propositions, we will gain a greater understanding of the barriers and facilitators to shared understanding; theoretical propositions also allow us to explore direct and indirect causal pathways for successful implementation of our interventions [24].

Second, implementation science theories should acknowledge that communication is multi-layered, identify at least a few layers, and posit how identified layers might affect the development of shared understanding required for successful implementation intervention success. One layer might consist of the tone of voice, words that are used, and body language for example, which all contribute to the meaning of the message and how it is acted upon. The medium by which a message is conveyed contributes additional complexity and another layer to communication [61]. These are basic communication characteristics, yet their influence on the development of shared understanding has not been established.

Third, while many implementation theories describe the environment or context in which an intervention takes place and its effect on implementation intervention success, the effect of the work environment on communication specifically is not well understood. Thus we recommend that implementation science theories acknowledge that communication occurs in a social context, providing a frame of reference for both individuals and groups. Going back to our example at the start of this paper, for individuals, high clinician workload and multiple interruptions (both contextual features of the ICU environment) may have disrupted cognitive processes needed to understand the clinical intervention and why it was being done. The social context also consists of characteristics such as social hierarchies and group dynamics which also influenced the development of shared understanding and contributed to the lack of consensus on how the intervention was to be carried out.

Fourth, we recommend recognizing the unpredictability of communication (and healthcare processes in general) because thinking of communication solely as information transfer or exchange misrepresents reality. Healthcare organizations are often conceptualized as stable systems whose function mimic machines (i.e., input-throughput-output) when in reality they are complex adaptive systems “beset with uncertainty” [64]. Clinicians engage in non-linear, unpredictable interactions, adapting to situations as they arise, thinking and acting simultaneously to find solutions to patient care problems [65]. Healthcare work is self-organized and occurs through random conversations to accomplish that work [56]. Structured communication tools such as SBAR are not designed to capture random conversations and while such tools are helpful in focusing on a specific patient care need, [66] they may not be effective for developing shared understanding because they do not contribute to the creation of meaning through unpredictable dialogue [55].

Finally, we recommend that research on the importance of communication for successful implementation be done. To achieve this recommendation, colleagues interested in implementation science should engage with researchers who study communication or at least become more familiar with the communication literature. In this paper, we have dichotomized the communication literature broadly as transactional versus transformational. However, there are interesting nuances within each category that may provide additional explanatory power for implementation phenomena. For example, conversation analysis (or “talk-in-interaction”) is used to reveal how, through language, individuals create meaning and action [67].

### Summary

Implementation science literature has depended on a communication paradigm in which communication is conceptualized as a transactional process (when communication has been mentioned at all), thereby ignoring a key contributor to implementation intervention success. In this paper, we have described another communication paradigm to help move implementation science forward. When conceptualized as a transformational process, the focus of communication moves to shared understanding and relationship development and thus is grounded in human interactions and the way we go about constructing knowledge. In healthcare, implementation intervention success depends on the co-creation of knowledge between the various disciplines providing patient care, but little attention has been paid to the details of how this occurs. Instead of hiding in plain sight, we suggest explicitly acknowledging the role that communication plays in our implementation efforts. By using both paradigms, we can investigate when communication facilitates implementation, when it does not, and how to improve it so that our implementation and clinical interventions are embraced by clinicians and patients alike.
